# Niagara Falls, 1899

**Published:** 1899-09

**Authors:** 


### Editorial.

### NIAGARA FALLS, 1899.

THE anticipation of a large meeting of dentists has been always realized at Niagara, and the present year has proved no exception, for over four hundred gathered there to meet with the national and allied educational conventions.

To those who were in attendance for years at the old American Dental Association there is much that endears this spot to memory. It was here that the earlier and best work of this organization was accomplished, and it was here that many, whose voices are forever silenced, helped to lay broad the foundations for the dentistry of this period. The older members, therefore, return to it with feelings of historic reverence, and while they enjoy, in common with all others, the great charm of Falls, Rapids, and the bewildering Gorge, they also come to it with feelings others may not share,—feelings that unite the past with the present.

While the foregoing is true, the Niagara of the present year cannot be regarded as entirely satisfactory. Formerly the place was the quiet resort of travellers, and a few days there could be thoroughly enjoyed. This is all changed, for it has become the working place of conventions at this season of the year and the railroads pour excursionists into the city by thousands. The result is that the old time comfort has disappeared, and in its place wrangling over accommodations, supplemented with difficulties at the table. There appeared to be no attention paid to contracts for rooms by letters. In no case, so far as the writer's observation went, was the promise "of a comfortable room" lived up to, and confusion prevailed upon the arrival of every train. This constitutes the disagreeable side of Niagara as a place of meeting in August.

While the place of meeting next year is at this time unknown to the writer, he leaving before the close, it is evident that August is not the proper time of year for a meeting of this character. The argument has always been that dentists usually take August as a vacation month. While this is true of those in large cities, it is not true of others, and a change to an early spring month is most desirable.

The National Dental Association had young blood at its head the present year, with the result that more papers and of better quality were presented than at any other meeting in the writer's memory. The conflict of interest with duty made impossible attendance upon all the sessions, as the meetings of the National Association of Dental Faculties interfered.

The middle West was represented by some of its strongest men, and while they presented views not in accord with recognized thoughts and observation, they were none the less acceptable, for they gave evidence of a breaking away from the shackles of authority, always refreshing, although they may not always be based on a reliable foundation.

The writer was not there long enough to learn of the final disposition of the surplusage of papers. It is equally unfortunate to have a superabundance in this direction as it is to have a paucity of material, for it is discouraging to workers not to be able to read their productions, and this may result, as upon previous occasions, in future weakness.

The outlook for the National Dental Association from the stand-point of this meeting is encouraging. Representatives from all portions of the United States and Canada were present. Dr. N. S. Jenkins, of Dresden, Germany, added much to the interest in presenting his porcelain inlay work, which has come to be regarded, by those familiar with it, as superior to other methods.

The action of the National Association of Dental Faculties last year, in suggesting the appointment of committees in various countries to work in connection with it, resulted in the visit of several gentlemen from different sections of Europe, thus giving almost an international character to this year's gathering.

It is to be hoped that the proceedings of the National will be issued at an earlier date than the volume of last year, they not appearing until nearly the time of the succeeding meeting.

Great credit is due all concerned for the success attained at the session of the National Association, and it is felt that, as far as the meetings were concerned, Niagara Falls will be remembered with satisfaction and its work be worthy of special remembrance.

